# Real-Time Sequence-Validated Loop-Mediated Isothermal Amplification Assays for Detection of Middle East Respiratory Syndrome Coronavirus (MERS-CoV)

**DOI:** 10.1371/journal.pone.0123126

**Published:** 2015-04-09

**Authors:** Sanchita Bhadra, Yu Sherry Jiang, Mia R. Kumar, Reed F. Johnson, Lisa E. Hensley, Andrew D. Ellington

**Affiliations:** 1 Institute for Cellular and Molecular Biology, Center for Systems and Synthetic Biology, Department of Chemistry and Biochemistry, University of Texas at Austin, Austin, Texas, United States of America; 2 Emerging Viral Pathogens Section, National Institute of Allergy and Infectious Diseases, National Institutes of Health, Fort Detrick, Maryland, United States of America; 3 Integrated Research Facility, National Institute of Allergy and Infectious Diseases, National Institutes of Health, Fort Detrick, Maryland, United States of America; National Cheng Kung University, TAIWAN

## Abstract

The Middle East respiratory syndrome coronavirus (MERS-CoV), an emerging human coronavirus, causes severe acute respiratory illness with a 35% mortality rate. In light of the recent surge in reported infections we have developed asymmetric five-primer reverse transcription loop-mediated isothermal amplification (RT-LAMP) assays for detection of MERS-CoV. Isothermal amplification assays will facilitate the development of portable point-of-care diagnostics that are crucial for management of emerging infections. The RT-LAMP assays are designed to amplify MERS-CoV genomic loci located within the open reading frame (ORF)1a and ORF1b genes and upstream of the E gene. Additionally we applied one-step strand displacement probes (OSD) for real-time sequence-specific verification of LAMP amplicons. Asymmetric amplification effected by incorporating a single loop primer in each assay accelerated the time-to-result of the OSD-RT-LAMP assays. The resulting assays could detect 0.02 to 0.2 plaque forming units (PFU) (5 to 50 PFU/ml) of MERS-CoV in infected cell culture supernatants within 30 to 50 min and did not cross-react with common human respiratory pathogens.

## Introduction

Coronaviruses (CoV) are large positive-stranded RNA viruses whose genomes range between ~27 to ~31 kb in size. They pose a continuing challenge to human health because their ability to infect a wide variety of organisms (including avian and mammalian species) fosters rapid evolution of their genomic RNA by recombination [1]. This may generate viral strains that are more virulent or recalcitrant to therapeutic interventions. Four human coronaviruses (hCoV-229E, hCoV-NL63, hCoV-OC43 and hCoV-HKU1) are in global circulation and cause respiratory infections typically characterized as the common cold [2]. A novel fifth hCoV, termed severe acute respiratory syndrome coronavirus (SARS-CoV), emerged during 2002 to 2003, and affected nearly 8000 people with a 10% mortality rate [2].

Another novel hCoV, Middle East respiratory syndrome coronavirus (MERS-CoV), was identified in 2012 in a Saudi Arabian patient who died from a severe respiratory illness termed the Middle East respiratory syndrome (MERS) [3, 4]. As of October 2, 2014, 853 laboratory-confirmed cases of MERS-CoV infections with 301 deaths have been reported globally to the World Health Organization (WHO) [5, 6]. Since March 2014, the infection frequency rapidly increased, with 647 cases having been reported within the past six months [5, 7]. Infections have been catalogued in the Middle East (Jordan, Kuwait, Oman, Qatar, Saudi Arabia and the United Arab Emirates), Africa (Egypt and Tunisia), Europe (France, Germany, Greece, Italy and the United Kingdom), Asia (Malaysia and Philippines) and North America (the United States of America) [8–15]. Numerous clustered cases in Europe and the Middle East indicate that human to human transmission is occurring via droplets or direct contact in healthcare environments, households, and the workplace [16–18]. Clinical presentation of MERS-CoV infection ranges from asymptomatic (21%) or mild symptoms (5%) to very severe pneumonia with acute respiratory distress syndrome, septic shock, and potentially multi-organ failure resulting in death (62%) [7]. While currently MERS-CoV apparently has limited pandemic potential, a human epidemic might still result from sustained transmission in animal reservoirs with sporadic human spill-overs and sustained human-to-human transmission [19, 20].

Due to the rapid rise in infection frequency, global spread, and high mortality rate it is critical to deploy point-of-care (POC) diagnostic devices to aid disease monitoring and management in the afflicted areas [21]. Corman *et al*. have reported diagnostic real-time reverse transcription polymerase chain reaction (RT-PCR) assays for qualitative and quantitative detection of MERS-CoV that are available in the RealStar kit (Altona Diagnostics GmbH, Hamburg, Germany) [2, 22]. These assays target regions in and around the viral genes open reading frame (ORF)1a, ORF1b and E. While these assays have been expertly designed and validated their execution demands expensive instrumentation and a dedicated laboratory environment with technically skilled operators. These characteristics are unsuitable to on-site disease tracking, which is critical for monitoring worldwide emerging infections [21]. Serological assays have also been developed including conventional immunofluorescence assays using virus-infected cells as well as Vero cells expressing the MERS-CoV N and S proteins [23], but serological distinction of different hCoVs remains challenging [24].

Isothermal amplification methods and probes have been widely used for nucleic acid diagnostics [25–27]. Among these, loop-mediated isothermal amplification (LAMP) [28] is ideally suited for field-based nucleic acid diagnostics due to minimal requirements for instrumentation, high sensitivity, and rapid results [29, 30]. Standard LAMP is a four-primer technique of auto-cycling strand displacement DNA synthesis mediated by the large fragment of *Bst* DNA polymerase using four primers designed to recognize six sequences (F3, F2, F1, B3, B2 and B1) in the target DNA [28]. Inner primers (FIP and BIP) composed of antisense and sense target sequences (F1c-F2 and B1c-B2, respectively) initiate DNA synthesis. The newly synthesized strands are released following strand displacement DNA synthesis primed by the outer primers (F3 and B3). The displaced strand can be primed by a second set of inner and outer primers and following extension yields a DNA product with a stem-loop structure. In subsequent cycles inner primers hybridize to the loops and initiate strand displacement DNA synthesis. This continuous amplification process ultimately leads to the accumulation of as many as 10^9^–10^10^ copies of the target within an hour. Inclusion of two additional primers that bind the two loop regions (resulting in a six-primer LAMP reaction) can significantly accelerate the amplification reaction [31]. The final LAMP reaction product consists of a DNA concatamer in which loops interspersed between alternating inverted repeats of the target generate a cauliflower-like structure.

However, like most isothermal amplification reactions LAMP has a propensity to produce spurious amplicons. Nucleic acid probes that hybridize to the transiently single-stranded LAMP loop sequences have been previously reported to enhance assay specificity by allowing real-time sequence validation of LAMP amplicons. Examples include fluorescence resonance energy transfer (FRET) signals that arise when a pair of oligonucleotides labeled with a donor (fluorescein) or an acceptor (LC Red 640) fluorophore [32] bind simultaneously to LAMP loops. In another strategy, LAMP reactions containing both the amplicon of interest (target) and a closely related internal amplification standard (competitor) are probed with the ‘alternately binding quenching’ probe (ABQprobe) that can bind to the LAMP loops of both the target and the competitor. The 5’-end of the ABQprobe is labeled with the green fluorophore BODIPY FL that is quenched by electron transfer to guanosine residues in the target LAMP loops. In contrast, the probe fluorescence intensity remains high upon binding the internal competitor amplicons in which these guanine residues are replaced with cytosine. Thus the fluorescence intensity of the ABQprobe reflects the ratio of LAMP products arising from the target and competitor [33].

However, both methods suffer from potential drawbacks. The significant overlap in the binding site of the FRET probes and the loop primers can reduce the efficiency of probe and primer binding and thereby result in a competition that slows signal generation. Co-amplification of an internal competitor for detection using the ABQprobe can also decrease assay sensitivity. To overcome this difficulty, we have also exploited loop regions in developing novel, real-time, sequence-specific signal transducers termed one-step strand displacement (OSD) probes that can be used for real-time sequence validation of LAMP amplicons [34]. OSD probes are partial DNA duplexes whose longer, fluorophore-labeled strands hybridize to the target amplicons via toehold-mediated strand displacement reactions [35–37]. The ensuing release of the shorter, quencher-bearing oligonucleotides from the fluorescent probes results in fluorescence accumulation during the amplification reaction; the more correct loops that arise, the more strand displacement reaction and thus the more fluorescent probes that are released.

The thermodynamic properties of the OSD probes render them particularly sensitive to the detection of even single nucleotide mismatches in the target amplicons [37–39]. Furthermore, unlike both the previously reported methods that merely mirror the accumulation of amplicons OSDs are readily programmable for higher order functions such as signal amplification, signal integration, and signal display via analytical methods as different as fluorimetry, colorimetry, and electrochemistry [39].

Additionally, in our assay system one loop region is dedicated to priming, thus improving the speed and specificity of LAMP, while the other loop region is dedicated to an OSD probe. This so-called asymmetric five-primer LAMP is significantly faster than standard four-primer LAMP since the additional loop primer is generating more amplicons that can be probed by the OSD probe. These innovations are now applied to MERS-CoV detection by reverse transcription (RT)-LAMP, leading to the identification of as few as 0.02 to 0.2 plaque-forming units (PFU) (5 to 50 PFU/ml) of MERS-CoV virions in culture supernatants of infected cells.

Overall, the sequence-validated OSD-RT-LAMP assays should prove conducive to the development of POC devices. The OSD probes can be readily engineered for signal transduction to multiple readout platforms including colorimetry and electrochemistry. This programmability will be tremendously useful for rapid and seamless integration of the assays into any of a variety of signal detection devices, both for monitoring MERS and other diseases.

## Results and Discussion

### Assay and primer design

As a first step, primers for standard four-primer LAMP were designed to target three MERS-CoV genetic loci (**S1A and S1B Fig**). The WHO laboratory testing guidelines specify that at least two separate genetic loci of MERS-CoV must be amplified for definitive diagnosis [40]. To meet these criteria three genes were targeted to enable conclusive MERS-CoV diagnosis via isothermal amplification. Twenty one complete MERS-CoV genome sequences were aligned along with phylogenetically-related CoV species including Bat-HKU5-1, SARS-CoV, hCoV-229E, hCoV-HKU1, hCoV-NL63 and hCoV-OC43 (Table 1) [1, 41, 42]. Three genomic regions in and around the MERS-CoV genes ORF1a, ORF1b and E were chosen for primer design based on their sequence identity within the MERS-CoV strains and significant sequence divergence from the related CoV species. These genetic loci have been previously used successfully as targets for RT-PCR-based MERS-CoV diagnostic assays and have been recommended by the WHO for laboratory testing of MERS-CoV [2, 23, 40]. LAMP primer design was constrained to include at least a 30 bp gap between the F1 and F2 as well as between the B1 and B2 priming sites. These gaps will become part of the LAMP amplicon loop structures and were included to allow subsequent priming by ‘loop’ primers and detection via the OSD probes.

**Table 1:** Primer and probe sequences for MERS-CoV asymmetric five-primer OSD-RT-LAMP assays.

ORF1a.55	F3^a^	TTATGCAAACATAGTCTACGAG
	B3	CGCAAAGTTAGAAAGTGATGG
	FIP	AAGCATTAGTGGGGGCAAGCCCCACTACTCCCATTTCG
	BIP	ATGCGCACTACACATACTGATATTTGTACAATCTCTTCACTACAATGA
	LP	GGTGTCTACATTAGTATGTCACTTGTATTAG
	OSD-F	/56FAM/ CGA AGC CAA TTT GCA ACT GCA ATC AGC GCT GAG/3InvdT/
	OSD-Q	ATTGCAGTT GCAAATTG GCT TCG/3IABkFQ/
ORF1b.59	F3	ACAGTTCCTGGATATCCTAAG
	B3	CTCAGTGTCTACAACACCA
	FIP	AGCACCCTCAACATCGAAGCACTCGTGAAGAGGCTGTA
	BIP	TGCTTCCCGTAATGCATGTGGACTGGCTGAACAACAAAGT
	LP	CTA TCC AGC TTC GAA CTT GCC T
	OSD-F	/56FAM/CAC ACC AGT TGA AAA TCC TAA TTG TAG AGG CAC ATT GGT G/3InvdT/
	OSD-Q	CTCTACAATTA GGATTTTCAACTG GTGTG/3IABkFQ/
UpE.9	F3	AGTAAGATTAGCCTAGTTTCTGT
	B3	TCCATATGTCCAAAGAGAGAC
	FIP	GAGGAACTGAATCGCGCGTTGACTTCTCCTTAAACGGCA
	BIP	TTCACATAATCGCCCCGAGCTAATGGATTAGCCTCTACACG
	LP	GCAGGCACGAAAACAGTGGAAACAT
	OSD-F	/56FAM/TCGCTTATCGTTTAAGCAGCTCTGCGCTACTATGGGTCC/3InvdT/
	OSD-Q	TAGCGCAGAGCTGCTTAAACGATAAGCGA/3IABkFQ/

^a^ F3: forward outer primer, B3: reverse outer primer, FIP: forward inner primer, BIP: reverse inner primer, LP: loop primer, OSD-F: fluorophore strand of the OSD probe, OSD-Q: quencher strand of the OSD probe. Accession numbers of the MERS-CoV and related coronavirus genomic sequences that were analyzed for primer design are as follows: KC164505.2, JX869059.2, KC667074.1, KC776174.1, KF192507.1, KF186567.1, KF186566.1, KF186565.1, KF186564.1, KF600645.1, KF600644.1, KF600627.1, KF600612.1, KF600652.1, KF600630.1, KF600647.1, KF600651.1, KF600632.1, KF600620.1, KF600613.1, KF600628.1, KF514433.1, KF430201.1, JX504050.1, NC_005147.1, NC_004718.3, EF065509.1.

Four sets of inner and outer primers specific to each of the MERS-CoV targeted regions were then tested in end-point LAMP assays for their ability to amplify cloned gBlocks representing DNA copies of the MERS-CoV target regions. One nanogram of plasmid DNA with or without gBlock inserts was amplified in standard four-primer LAMP reactions incubated for 90 min at 65 °C. Following amplification, amplicons were analyzed by agarose gel electrophoresis. Two primer sets for each targeted region were chosen for further analysis based on their ability to yield a characteristic ladder-like pattern of concatameric LAMP amplicons only in the presence of template (data not shown). The amplification kinetics of these four-primer LAMP systems was then analyzed in real-time LAMP reactions containing titrating concentrations of plasmid DNA templates and EvaGreen, the fluorescent intercalating dye (**S2 Fig**). Ultimately, the primer sets ORF1a.55, ORF1b.59 and UpE.9 demonstrated target-dependent amplification kinetics and generated minimal spurious amplicons in the absence of specific templates and were chosen for further assay development (**Table 1**).

### Harnessing LAMP amplicon loops for MERS-CoV sequence-validation and increased speed of detection

We then designed an additional single loop primer and OSD probe to augment each of the primer sets and compared the kinetics of MERS-CoV-specific, real-time, asymmetric five-primer OSD-LAMP to that of the standard four-primer real-time OSD-LAMP. Different copy numbers of MERS-CoV surrogate templates (cloned into plasmids) were amplified using standard four-primer OSD-LAMP or asymmetric five-primer OSD-LAMP assays at 65 °C. The reaction kinetics were measured in real-time by quantitating fluorescence accumulation using the Roche LightCycler 96 real-time PCR machine. Our results demonstrated that the asymmetric five-primer OSD-LAMP assays significantly improved the speed of amplicon generation. While the standard four-primer OSD-LAMP assay using the primer set ORF1a.55 could detect 2000 molecules within about 72 min (Cq 23.74), the asymmetric five-primer OSD-LAMP could achieve the same result within 30 min (Cq 10.35). Similarly, the standard four-primer OSD-LAMP assay with ORF1b.59 required 45 min (Cq 15.24) to detect 2000 molecules of template while in the asymmetric five-primer format a similar limit of detection (LOD) was achieved within 24 min (Cq 7.96). Likewise, the standard four-primer OSD-LAMP assay with UpE.9 primer required 81 min (Cq 26.81) for detection of 2000 template molecules and the asymmetric five-primer assay required only 27 min (Cq 9.3).

The LODs for these assays with plasmid templates were also determined (**S3 Fig**). All three MERS-CoV amplicons could be detected down to 20 copies of the corresponding surrogate DNA templates. While ORF1b.59 and UpE.9 assays could detect 20 molecules within ~30 min, the ORF1a.55 assay required almost 1 h to achieve a similar LOD. However, the ORF1a.55 LAMP assay also demonstrated the highest sensitivity and could often detect down to 2 template DNA copies.

These results demonstrate that we have successfully developed and validated isothermal assay methods for detection of the emerging human coronavirus MERS-CoV. Our assay is based upon a previously described molecular amplification method, LAMP [28], but goes well beyond this method by incorporating real-time sequence-specific signal transducers (OSD probes) that are based upon nucleic acid strand displacement methods originally developed for DNA computation [39, 43–47].

### Design of a one-pot RT-LAMP assay for MERS-CoV RNA detection

After optimizing the asymmetric five-primer OSD-LAMP assays for detection of MERS-CoV-specific DNA amplicons, we sought to develop an asymmetric five-primer, OSD-transduced, one-pot RT-LAMP assay system suitable for detection of MERS-CoV viral RNA. For this purpose, T7 RNA polymerase-driven transcription templates containing MERS-CoV-derived sequences were generated by PCR-mediated amplification of the cloned MERS-CoV surrogate gBlock sequences. Following *in vitro* transcription and subsequent removal of the DNA transcription templates by DNase I digestion, the transcripts were purified by denaturing polyacrylamide gel electrophoresis. Known amounts of MERS-CoV-specific or non-specific RNA templates were then used to optimize the buffer and enzymatic conditions for one-pot RT-LAMP assays (**S4 Fig**). Our results indicate that while *Bst* 2.0 DNA polymerase demonstrated significant RT-LAMP activity alone (based on its inherent reverse transcriptase activity [48]), the reaction speed was substantially improved by incorporation of AMV reverse transcriptase (AMV RT) in the reaction. AMV RT is also known to generate significant amounts of cDNA at temperatures as high as 65 °C [49]. Furthermore, while LAMP amplification of DNA templates was efficient in 1X Isothermal buffer, one-pot RT-LAMP performance was improved by using a buffer system composed of 1X Thermopol buffer and 0.5X AMV RT buffer. The speed of the assay was further improved by reducing the betaine concentration from 1 M to 0.4 M in the reactions. Betaine is an amino acid analogue that reduces the melting temperature of nucleic acids and diminishes the influence of base pair composition on thermal melting transitions [50]. Reducing betaine concentrations may improve amplification due to a combination of factors. Betaine has previously been used to improve polymerase-mediated amplification of structured GC-rich templates and to improve PCR specificity [51, 52]. The enzymatic activities of *Bst* 2.0 DNA polymerase and AMV RT during RT-LAMP may be more efficient at lower betaine concentrations. Similarly, some reports suggest that varying betaine concentrations uniquely impacts RNA tertiary structures (and thus potential availability for reverse transcription) depending on the identity of the RNA as well as the concentration and identity of cations [53].

**Fig 1:**
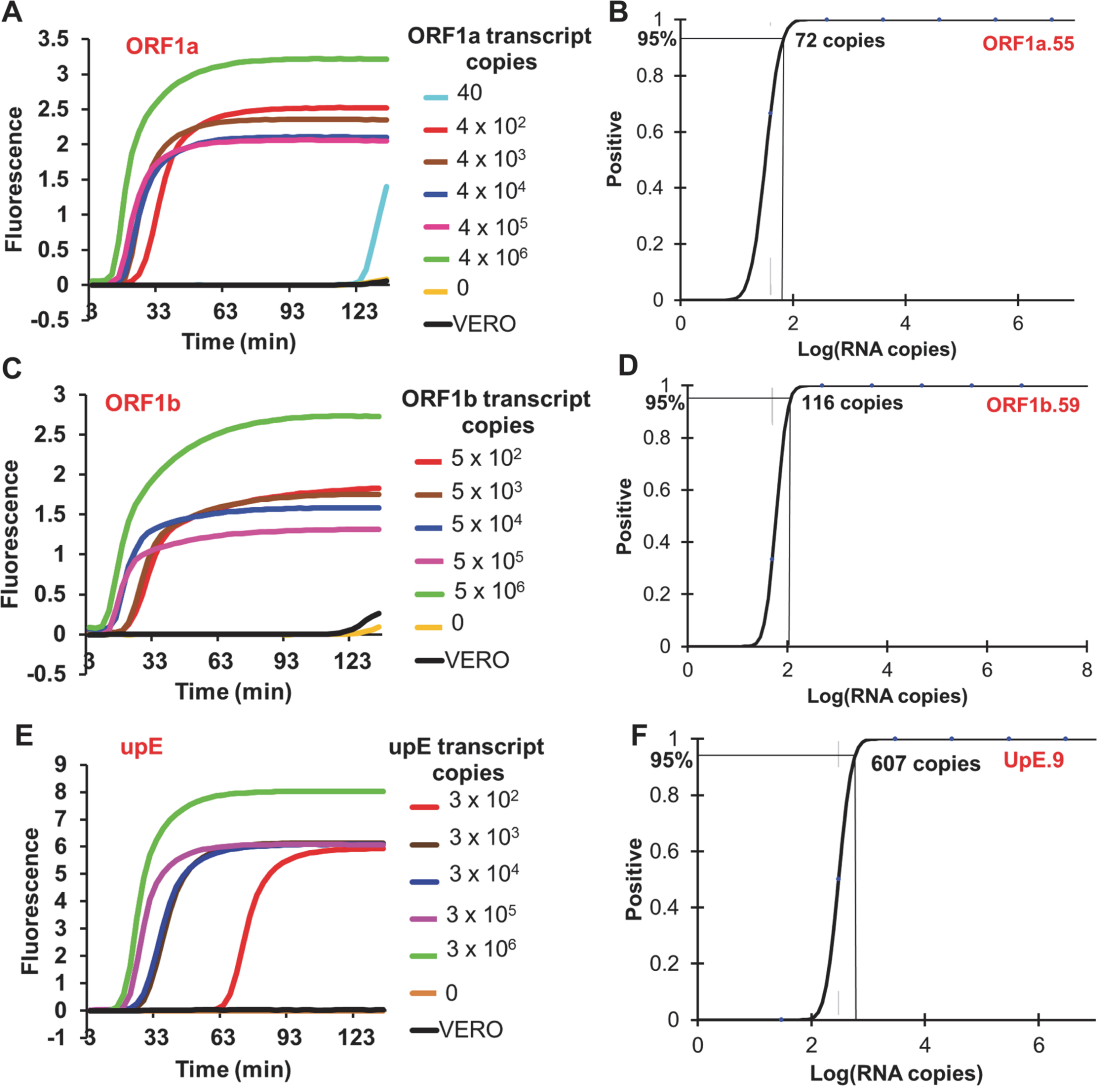
Determination of the technical LOD of one-pot asymmetric five-primer OSD-RT-LAMP assays designed for MERS-CoV detection. Technical LOD of the ORF1a.55, ORF1b.59 and UpE.9 asymmetric five-primer LAMP primer sets was determined by amplification of specific *in vitro* transcribed MERS-CoV RNA segments. Representative amplification curves of the ORF1a.55, ORF1b.59 and UpE.9 assays are depicted in panels A, C and E, respectively. Probit regression analysis plots with the calculated LOD of the ORF1a.55, ORF1b.59 and UpE.9 assays are depicted in panels B, D and F, respectively. Samples labeled ‘VERO’ consists of RNA extracted from uninfected Vero cell culture supernatants.

Different copy numbers of *in vitro* transcribed RNA templates were then evaluated using the optimized ORF1a.55, ORF1b.59 and UpE.9 one-pot RT-LAMP assays. EvaGreen used in the optimization reactions was replaced with 50 nM of the fluorophore-labeled OSD probe strand (pre-annealed with a 5-fold excess of the quencher strand) to allow real-time, sequence-specific monitoring of amplicon accumulation. ORF1a.55, ORF1b.59, and UpE.9 assays could detect down to 72, 116 and 607 copies, respectively, of *in vitro* transcribed MERS-CoV RNA sequences within 30 to 65 min of amplification (**Fig 1**), as opposed to 20 copies of plasmid DNA. The higher LOD of RT-LAMP assays compared to LAMP assays performed using DNA templates is likely due to reductions in the efficiency of reverse transcription [49] in the context of the high temperatures during one-pot RT-LAMP, just as transcription yields are also known to affect the limit of RNA quantitation by real-time PCR [54, 55]. A previous report demonstrated that one-pot RT-LAMP was very inefficient at digital RNA quantitation and generated counts that were only 2% of the expected value [56]. In contrast, digital LAMP assays without RT reflected 62% of the expected DNA concentration. The authors were able to improve their RT-LAMP absolute quantification efficiency from 2% to 23% by using a two-step assay in which reverse transcription was first performed using a more efficient reverse transcriptase, addition of only the BIP primer during reverse transcription, and introduction of RNase H to break up the DNA:RNA hybrids to facilitate primer annealing and subsequent LAMP amplification of the resulting single-stranded cDNA.

### Sensitive detection of MERS-CoV virions from cell culture supernatants

**Fig 2:**
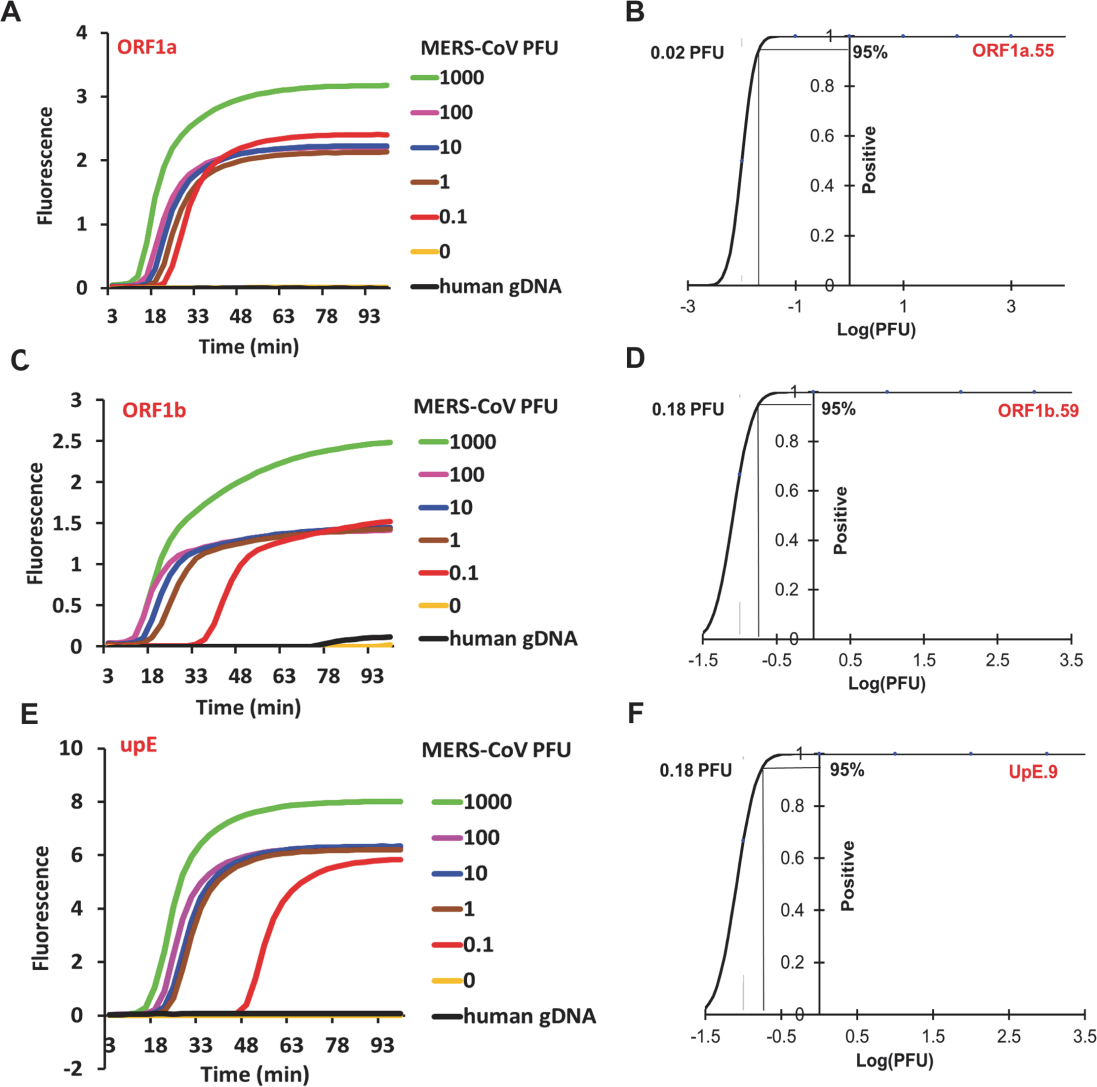
Detection of MERS-CoV in cell culture supernatants using one-pot asymmetric five-primer OSD-RT-LAMP assays. MERS-CoV genomic RNA extracted from different amounts of MERS-CoV plaque forming units were amplified by asymmetric five-primer OSD-RT-LAMP assays using the ORF1a.55, ORF1b.59 and UpE.9 primer sets. Representative amplification curves of the ORF1a.55, ORF1b.59 and UpE.9 assays are depicted in panels A, C and E, respectively. Probit regression analysis plots with the calculated LOD of the ORF1a.55, ORF1b.59 and UpE.9 assays are depicted in panels B, D and F, respectively. Reactions that were not seeded with specific templates or that contained human genomic DNA were used as negative controls.

To determine the efficacy of the one-pot OSD-RT-LAMP assays in detecting clinically relevant sources of MERS-CoV RNA we sought to amplify genomic RNA isolated from MERS-CoV virions. RNA was isolated from Trizol-inactivated MERS-CoV (Jordan n3/2012 strain)-infected Vero cell culture supernatants containing MERS-CoV virions along with defective interfering particles. Given the virion concentration of 3 x 10^6^ PFU/ml in the 3:1 diluted cell culture supernatants the virus genomic RNA concentration in the extracted RNA was found to be 3 x 10^4^ infectious genomes/μl. The MERS-CoV-specific asymmetric five-primer OSD-RT-LAMP assays did not generate significant signal when presented with human genomic DNA or RNA extracted from culture supernatants of uninfected Vero cells (**Figs 1 and 2**).

In the presence of serially-diluted MERS-CoV virion-derived RNA the ORF1a.55, ORF1b.59 and UpE.9 asymmetric five-primer OSD-RT-LAMP assays successfully detected as few as 0.02 to 0.2 PFU or 5 to 50 PFU/ml of MERS-CoV virions within 30 to 50 min (**Fig 2**). To obtain statistically accurate LOD measurements a series of dilutions containing decreasing numbers of template copies was analyzed in multiple parallel assays. The dilution series included both samples with high template concentrations (that always tested positive) and samples with very few template copies (such that some reactions failed to generate signals above background). At each template concentration the fraction of positive reactions compared to the total number of assays performed was subjected to Probit regression analysis. The number of template copies that could be detected with 95% certainty was considered to be the assay LOD. The fact that we were able to detect smaller numbers of particles than the previously determined RNA LOD would have indicated is likely due to the excess RNA originating from defective interfering particles.

Our methods compare well to previously reported, more cumbersome RT-PCR assays for MERS-CoV [2, 23]. The upE RT-PCR assay can detect 0.01 median tissue culture infectious dose (TCID_50_) of MERS-CoV per 5 μl (translating to 2 TCID_50_/ml or approximately 1.4 PFU/ml) with a technical LOD of 3.4 synthetic RNA molecules; in contrast our UpE.9 OSD-RT-LAMP assay can detect 50 PFU/ml of MERS-CoV with a technical LOD of 607 synthetic RNA molecules. The ORF1b RT-PCR assay can detect 0.1 TCID_50_ of MERS-CoV per 5 μl (translating to 20 TCID_50_/ml that is approximately equivalent to 14 PFU/ml) with a technical LOD of 64 synthetic RNA molecules. Compared to this, our ORF1b.59 OSD-RT-LAMP assay can detect 50 PFU/ml of MERS-CoV with a technical LOD of 116 synthetic RNA molecules. Finally, our ORF1a.55 OSD-RT-LAMP assay can detect 5 PFU/ml of MERS-CoV but displays a higher technical LOD (72 synthetic RNA copies) as compared to 4.1 synthetic RNA copies for the ORF1a RT-PCR assay.

Similarly, a different RT-LAMP assay for MERS-CoV detection has been described by Shirato *et al*. [57]. This assay was designed to amplify MERS-CoV N gene sequence and was reported to detect as few as 3.4 copies of MERS-CoV RNA. However, amplicon detection was based on measuring an increase in turbidity or calcein fluorescence due to accumulation of pyrophosphate, a sequence-independent amplification by-product. Considering the fact that LAMP is notorious for generating spurious amplicons such indirect measures are fraught with the likelihood of generating false positive results. In fact the authors themselves noted that their primers generated significant quantities of non-specific amplicons after just 30 min of amplification.

An additional isothermal amplification assay based on the reverse transcriptase recombinase polymerase assay was described in 2013 for the N gene of MERS-CoV [58]. The authors reported a fluorimetric LOD of 21 synthetic RNA copies within 3 to 7 min and a detection capability for 3000 genome equivalents/ml of RNA extracted from MERS-CoV tissue culture supernatant. Although recombinase polymerase assay has been adapted to some systems such as paper-based devices [59], a microfluidic lab-on-a-foil [60], and digital slip chips [61] its operation requires a proprietary commercial kit that makes it difficult to reconfigure the assay. Moreover, the flexibility of design is limited by the fact that a probe with several modifications such as fluorophores, quenchers, and tetrahydrofuran labels must be purchased separately to obtain a template-specific signal. Finally, this probe only monitors amplicon accumulation while our RT-LAMP method enables robust signal integration and transduction.

Overall, while the RT-LAMP assays are 2- to 200-fold less sensitive than PCR, these differences are not clinically relevant since viral loads can be as high as 1–2 x 10^6^ copies/ml in the lower respiratory tract [62]. Similarly, a maximum viral load of 2691 RNA copies/ml in urine was observed on day 13 of infection prior to renal failure, while stool samples contained up to 1031 viral RNA copies/g. One of two oronasal swabs from day 16 of infection contained 5370 copies of viral RNA/ml. All of these viral loads should be well within the limits of detection we have demonstrated, and thus the simplicity and ease of detection for these different assays become the more relevant and significant comparators.

### Assay specificity

**Fig 3:**
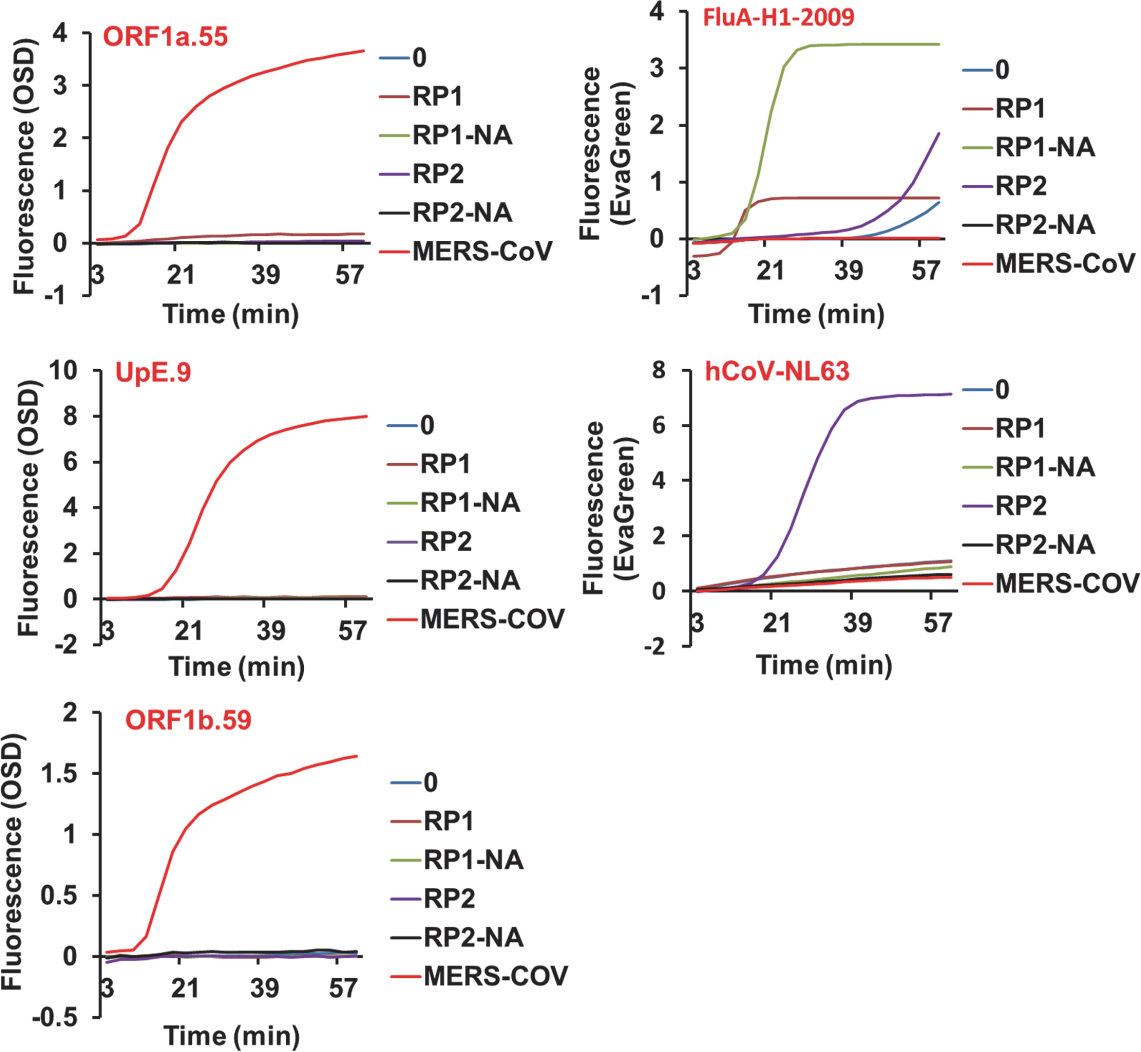
Specificity of MERS-CoV OSD-RT-LAMP assays. RP1 and RP2 refer to the unprocessed NATtrol multimarker respiratory panels. RP1-NA and RP2-NA refer to total nucleic acids extracted from RP1 and RP2, respectively. MERS-CoV refers to 1 x 10^4^ infectious genomic RNA extracted from tissue-culture-derived MERS-CoV virions while ‘0’ refers to amplification reactions that were not seeded with any template.

To obtain a more clinically relevant assessment of assay specificity the cross-reactivity of the MERS-CoV-directed asymmetric five-primer OSD-RT-LAMP assays was checked by amplifying the NATtrol multimarker controls RP1 and RP2 (Zeptometrix Corporation, Buffalo, New York, USA). These respiratory pathogen panels were formulated with chemically-inactivated organisms suspended in a purified protein matrix that acts as a surrogate for clinical specimens (**Table 2**). Aliquots of unprocessed RP1 and RP2 as well as total nucleic acids extracted from these panels were analyzed by MERS-CoV-specific OSD-RT-LAMP assays. Our results demonstrated that the ORF1a.55, ORF1b.59 and UpE.9 OSD-RT-LAMP primer sets did not cross-react with a broad range of human respiratory pathogens including other common hCoVs present in the RP1 and RP2 panels (**Fig 3**). To demonstrate the presence of amplifiable nucleic acids in the RP1 and RP2 panels LAMP primer sets described in literature were used to amplify Flu A-H1-2009 [63] and hCoV NL63 [64] as controls.

**Table 2:** Composition of the NATtrol respiratory agent panels.

**Target**			**RP Multi 1**	**RP Multi 2**
Adenovirus			Positive	Negative
Coronavirus 229E			Negative	Positive
Coronavirus HKU1			Negative	Positive
Coronavirus NL63			Negative	Positive
Coronavirus OC43			Negative	Positive
Human metapneumovirus			Positive	Negative
Human rhinovirus/enterovirus		Entero 1	Positive	Negative
		Entero 2	Positive	Negative
		HRV1	Positive	Negative
		HRV2	Positive	Negative
		HRV3	Positive	Negative
		HRV4	Positive	Negative
Influenza AH1-2009	Flu-A-H1-2009		Positive	Negative
Influenza AH1	Flu-A-H1-pan		Positive	Positive
Influenza AH3	Flu-A-H3		Positive	Negative
	Flu-A-pan1		Positive	Positive
	Flu-A-pan2		Positive	Positive
Influenza B			Negative	Positive
Parainfluenza virus 1			Positive	Negative
Parainfluenza virus 2			Negative	Positive
Parainfluenza virus 3			Negative	Positive
Parainfluenza virus 4			Positive	Negative
Respiratory Syncytial Virus			Negative	Positive
*Bordetella pertussis*			Negative	Positive
*Chlamydophila pneumonia*			Positive	Negative
*Mycoplasma pneumonia*			Positive	Negative

Although highly conserved regions of the MERS-CoV genome were originally chosen as amplification targets the primer and OSD probe sequences were again compared to the most current MERS-CoV genomic sequence database using BLAST to confirm their ability to detect newly emergent MERS-CoV strains. While the nucleic acid components of the ORF1a.55 and ORF1b.59 assays continued to display a complete match with the latest NCBI sequence database, the UpE.9 F1 primer and the loop primer (LP) displayed single nucleotide mismatches with one new MERS-CoV genomic sequence each (GenBank accession numbers KJ156873.1 and KJ156881.1, respectively). To ensure that neither of these mutations could cause the UpE.9 OSD-RT-LAMP assay to falsely fail, we synthesized two mutated versions of the upE RNA template each bearing one of the single nucleotide changes. In parallel amplification reactions comparing the mutant versus the wildtype upE RNA neither of these mismatches was found to compromise the UpE.9 OSD-RT-LAMP assays (**S5 Fig**). This observation suggests that our OSD-RT-LAMP assays are extremely robust, especially when used in combination, for accurate MERS-CoV diagnostics. In contrast, the N gene LAMP primer set previously described [57] displayed mismatches to as many as 21 sequences out of the 88 MERS-CoV isolates analyzed *in silico*, and thus these primers might fail to detect strains bearing additional mutations.

The MERS-CoV-specific OSD-RT-LAMP assays developed by us offer several advantages. (1) Our method ensures diagnostic validity by using OSD probes for real-time sequence-specific transduction of (RT)-LAMP amplicons into fluorescence signal. (2) The ORF1a, ORF1b and upE-specific RT-LAMP primer sets that we report generate negligible non-specific amplicons (as evident from lack of EvaGreen signal in the absence of template (**S2 Fig**)) even after more than 2 h of amplification. (3) Our primer and probe sets lie in highly conserved regions of the MERS-CoV genome. Of the 21 primer binding sites and 3 probe binding sites employed in the ORF1a.55, ORF1b.59 and UpE.9 MERS-CoV assays only UpE.9 F1 and LP display single mismatches with one MERS-CoV genomic sequence each in the current database. Neither of these mismatches was found to compromise the OSD-RT-LAMP assay when tested using mutated synthetic RNA (**S5 Fig**). (4) In light of the WHO’s emphasis on amplification of at least two separate MERS-CoV genomic targets for definitive diagnosis we have developed sequence-validated isothermal assays specific for 3 separate regions of the MERS-CoV genome. These assays may be used in parallel for reliable diagnosis of MERS-CoV.

## Conclusion

Isothermal nucleic acid amplification assays are particularly conducive to the development of field-deployable POC diagnostic devices [65], and the MERS-CoV OSD-RT-LAMP assays can potentially be loaded on such POC devices, which in turn could be used for managing viral spread and other epidemiological studies [21]. LAMP reactions have not previously been adapted to POC devices because of background issues relating to spurious amplicon generation and assay variance. The application of OSD probes solves this problem while obviating the need for more complex methods such as the incorporation of modified bases [66]. Employment of the sequence-specific OSD probes not only allows real-time assay validation but also provides the potential advantage of signal integration. For instance, activation of OSD probes can be readily engineered to require concomitant strand displacement by two different target sequences. Combinatorial operation of the three MERS-CoV-specific OSD-RT-LAMP assays may be used to further bolster diagnostic validity. More generally, the OSD-RT-LAMP assays we have developed represent a significant diagnostic advancement, enabling one-pot execution and rapid (30 to 50 min) time-to-result. Furthermore, the well-defined OSD probe design rules and the extreme robustness of the optimized OSD-LAMP and OSD-RT-LAMP methodologies will facilitate quick development of new diagnostic assays designed to detect varied pathogens and nucleic acids.

## Materials and Methods

### Chemicals and reagents

All reagents and chemicals unless otherwise noted were obtained from Sigma Aldrich (St. Louis, MO, USA). All oligonucleotides and gBlocks were obtained from Integrated DNA Technologies (IDT, Corralville, IA, USA). The fluorophore-labeled oligonucleotides used for assembling the duplex OSD probe were designed to contain an inverted dT group at their 3’-ends to prevent polymerase-mediated extension **(Table 1)**. Oligonucleotides were resuspended at 100 μM concentration in TE (10:0.1, pH 7.5) buffer (10 mM Tris-HCl, pH 7.5, 0.1 mM EDTA, pH 8.0) and stored at -20 °C. The concentrations of the DNA and RNA suspensions were measured by UV spectrophotometry using the NanoDrop 1000 spectrophotometer (Thermo Scientific, Wilmington, DE, USA). All enzymes including *Bst* 2.0 DNA polymerase and AMV RT were obtained from New England Biolabs (NEB, Ipswich, MA, USA). Human genomic DNA was obtained from Promega (Madison, WI, USA).

### Cloning of MERS-CoV gBlocks and PCR amplification of transcription templates

All DNA PCR amplification reactions were performed using high-fidelity Phusion DNA polymerase (NEB), according to the manufacturer’s instructions. The gBlock double stranded DNA surrogates of MERS-CoV genetic loci were designed to include a T7 RNA polymerase promoter at their 5’-ends to enable subsequent transcription. These gBlocks were cloned into the pCR2.1-TOPO vector (Life Technologies, Carlsbad, CA, USA) by Gibson assembly [67] using the 2X mastermix (NEB) according to the manufacturer’s instructions. Cloned plasmids were selected and maintained in an *E*. *coli* Top10 strain. Plasmid minipreps were prepared from these strains using the Qiagen miniprep kit (Qiagen, Valencia, CA, USA). All gBlock inserts were verified by sequencing at the Institute of Cellular and Molecular Biology Core DNA Sequencing Facility.

For performing *in vitro* run-off transcription, transcription templates cloned in a pCR2.1-TOPO vector were amplified from sequenced plasmids by PCR using Phusion DNA polymerase. PCR products were verified by agarose gel electrophoresis and then purified using the Wizard SV gel and PCR Clean-up system, according to the manufacturer’s instructions (Promega).

### *In vitro* transcription

Some 1000 ng of double-stranded DNA transcription templates were transcribed using 100 units of T7 RNA polymerase (NEB) in 50 μl reactions containing 40 mM Tris-HCl, pH 7.9, 30 mM MgCl_2_, 10 mM DTT, 2 mM spermidine, 4 mM ribonucleotide mix (NEB), and 20 units of the recombinant ribonuclease inhibitor RNaseOUT (Life Technologies). Transcription was allowed to occur at 42 °C for 2 h. Subsequently the transcription reactions were incubated with 2 units of DNase I (Life Technologies) at 37 °C for 30 min to degrade the template DNA prior to RNA gel purification.

### Denaturing polyacrylamide gel electrophoresis and RNA gel purification

Denaturing 10% polyacrylamide gels containing 7 M urea were prepared using 40% acrylamide and bis-acrylamide solution, 19:1 (Bio-Rad) in 1X TBE buffer (89 mM Tris Base, 89 mM Boric acid, 2 mM EDTA, pH 8.0) containing 0.04% ammonium persulphate and 0.1% TEMED. An equal volume of 2X denaturing dye (7 M urea, 1X TBE, 0.1% bromophenol blue) was added to the RNA samples. These were incubated at 65 °C for 3 min followed by cooling to room temperature before electrophoresis. The gels were stained for 10 min with SYBR-Gold (Life Technologies) prior to visualization on the Storm Imager (GE Healthcare, Fairfield, CT, USA). For RNA purification, desired bands were excised from the gel and the RNA was eluted twice into TE (10:1, pH 7.5) buffer (10 mM Tris-HCl, pH 7.5, 1 mM EDTA, pH 8.0) by incubation at 70 °C and 1000 rpm for 20 min. Acrylamide traces were removed by filtering eluates through Ultrafree-MC centrifugal filter units (EMD Millipore, Billerica, MA, USA) followed by precipitation with 2X volume of 100% ethanol in the presence of both 15 μg GlycoBlue (Life Technologies) and 0.3 M sodium acetate, pH 5.2. RNA pellets were washed once in 70% ethanol. Dried pellets of purified RNA were resuspended in 0.1 mM EDTA and stored at -80 °C.

### Primer and OSD probe design

Twenty one complete MERS-CoV genome sequences were obtained from NCBI GenBank and aligned along with phylogenetically-related CoV species including Bat-HKU5-1, SARS-CoV, hCoV-229E, hCoV-HKU1, hCoV-NL63 and hCoV-OC43 using MUSCLE (Table 1) [1, 41, 42]. Three genomic regions in and around the MERS-CoV genes ORF1a, ORF1b and E were chosen for primer design. The Primer Explorer v4 primer design software (Eiken Chemical Co., Japan) was used for generating several potential LAMP primer sets composed of the outer primers F3 and B3 and the inner primers FIP and BIP. Primer design was constrained to include at least a 30 bp gap between the F1 and F2 as well as between the B1 and B2 priming sites. Primer specificity for all the sequenced MERS-CoV isolates and a corresponding lack of significant cross-reactivity to other nucleic acids of human or pathogenic origin was further assessed using NCBI BLAST [68, 69].

The fluorophore-labeled OSD strands were designed to bind between F2 and F1 sequences of ORF1a, between B2 and B1 sequences of ORF1b and between B2c and B1c sequences of upE. Their 3’ end was blocked with inverted dT to prevent extension by DNA polymerase. The quencher-labeled OSD strand was designed to be partially complementary to the fluorophore-labeled strand. The lengths of the two strands were designed to ensure that the duplex region displayed a ΔG < = -18 kcal/mole (calculated using the NUPACK software suite) at 60 °C with salt concentration mimicking that of 1X Isothermal buffer. Single-stranded toeholds at the 3’-end of fluorophore-labeled strands were designed to be 10 or 11 nucleotides long.

### LAMP assay with DNA template

Immediately prior to use plasmid DNA containing target or non-specific inserts were serially diluted in TE (10:0.1) buffer and used as templates in LAMP reactions. End-point LAMP assays for agarose gel electrophoretic analysis were typically set up on a cold block in 25 μl volume using 1 ng of template DNA in 1X Isothermal buffer (NEB; 20 mM Tris-HCl, 10 mM (NH_4_)_2_SO_4_, 50 mM KCl, 2 mM MgSO_4_, 0.1% Tween 20, pH 8.8 at 25°C) containing 0.4 mM dNTPs, 1M Betaine, 2 mM additional MgCl_2_, 0.8 μM each of FIP and BIP and 0.2 μM each of F3 and B3. The reactions were denatured at 95 °C for 5 min followed by rapid cooling on ice for 2 min. The reactions were briefly spun down to collect condensation and 8 units of *Bst* 2.0 DNA polymerase were added to the reactions that were then incubated at 65 °C for 90 min. Subsequently, the *Bst* 2.0 DNA polymerase was thermally denatured by incubation at 80 °C for 20 min. This precaution was taken to minimize the spread of LAMP amplicon contamination.

Real-time LAMP reactions monitored with EvaGreen (Biotium, Hayward, CA, USA) were assembled exactly as above with the addition of 1X EvaGreen. Typically double the reaction volumes were prepared and aliquoted into duplicate wells of a 96-well LightCycler 96 white PCR plate. The reactions were analyzed using the LightCycler 96 (Roche, Basel, Switzerland) that was set up to incubate the samples for 45 cycles of two-step incubations—step 1: incubation at 65 °C for 150 sec, step 2: incubation at 65 °C for 30 sec (total incubation time of 3 min / cycle unless otherwise indicated). EvaGreen signal was measured in the FAM channel during step 2 of each cycle. The 45 cycles of two-step amplification were followed by incubation of the reactions at 80 °C for 20 min in order to inactivate the *Bst* 2.0 DNA polymerase. Subsequently, amplicons were subjected to a melt analysis by incubation at 65 °C for 1 min followed by 0.1 °C/s incremental rise in temperature to 97 °C. Amplicon melting was monitored by measuring fluorescence at the rate of 10 readings per °C change in temperature. The resulting data was analyzed using the LightCycler 96 analysis software to generate Cq values for amplification and also to obtain the melting temperatures of LAMP amplicons detected by EvaGreen intercalation.

Real-time LAMP reactions monitored with OSD probes were assembled and analyzed exactly as above with the following changes. The annealed OSD probes were added to the LAMP reactions after the template:primer denaturation and annealing step had been performed. OSD probes were prepared by annealing the fluorophore and quencher oligonucleotides in a ratio of 1:5 in 1X Isothermal buffer. Annealing was performed by denaturing the oligonucleotide mix at 95 °C for 1 min followed by slow cooling at the rate of 0.1 °C/s to 25 °C. Excess annealed probe was stored at -20 °C. Annealed OSD probes were added to the LAMP reactions at a final concentration of 50 nM of the fluorophore-bearing strand.

Asymmetric LAMP reactions with an additional loop primer were assembled as detailed above but with higher primer concentrations. The reactions included 1.2 μM each of FIP and BIP, 0.3 μM each of F3 and B3 and 0.6 μM of the loop primer.

### One-pot RT-LAMP assay

RNA samples were serially diluted in TE (10:0.1) buffer immediately prior to amplification by RT-LAMP. For amplification of RNA by one-pot RT-LAMP reaction 4 μl aliquots of the RNA template were mixed with 1.12 mM dNTPs, 1.6 μM each of FIP and BIP and 0.4 μM each of F3 and B3. For asymmetric five-primer RT-LAMP reactions 0.8 μM of the loop primer was also included. This RNA: primer mix was then incubated at 95 °C for 1 min followed by quick cooling on ice for at least 2 min. The RT-LAMP assay was assembled for a final volume of 25 μl containing 1X Thermopol buffer (NEB; 20 mM Tris-HCl, 10 mM (NH_4_)_2_SO_4_, 10 mM KCl, 2 mM MgSO_4_, 0.1% Triton X-100, pH 8.8 at 25°C), 0.5X AMV RT buffer (NEB; 25 mM Tris-HCl, 37.5 mM potassium acetate, 4 mM magnesium acetate, 5 mM DTT, pH 8.3 at 25 °C), 50 nM of the OSD probe fluorophore-labeled strand, 2 mM additional MgCl_2_, 0.4 M Betaine, 8 units of *Bst* 2.0 DNA polymerase and 2 units of AMV RT. This enzyme and probe mix was added to the annealed RNA, primer and dNTP mix followed by analysis on the LightCycler 96 as detailed above.

Un-processed NATtrol RP multimarker panels as well as total nucleic acids extracted from these panels were diluted 1:10 in TE (10:0.1) and 4 μl aliquots were immediately used for analysis. RT-LAMP assays were performed using the same procedure as detailed above with the exception that 1X EvaGreen was used instead of OSD probes for detection of hCoV NL63 and Flu A-H1-2009 amplicons.

### Statistical analysis

Probit regression analysis for determining the limits of detection of the ORF1a.55, ORF1b.59 and UpE.9 OSD-RT-LAMP assays were performed using the ‘Dose Effect Analysis’ module in the XLSTAT statistical add-in software (Addinsoft, New York, NY, USA) for Microsoft Excel.

### Virus propagation

MERS-CoV Jordan n3/2012 isolate was propagated in Vero cells (obtained from ATCC, catalog number CCL81) at a multiplicity of infection of 0.1 for 3–4 days post-inoculation until cytopathic effect encompassed 75–80% of the Vero monolayer. Vero cells were maintained in Dulbecco’s Modified Eagle’s medium (HyClone, Logan, UT) supplemented with 10% fetal bovine serum (Sigma Aldrich) and 1% penicillin/streptomycin at 37 °C with 5% CO_2_. Virus was recovered by removal of the media and centrifugation at 1200xg for 10 min at 4 °C. Supernatant was aliquoted, frozen and titered by limiting dilution plaque assay on Vero cells using an overlay of 0.8% tragacanth in Eagle’s Minimum Essential Medium containing 2% fetal bovine serum and 1% penicillin/streptomycin for 3–5 days. The resulting plaques were enumerated after staining the cells for 30 min with crystal violet containing 20% neutral buffered formalin.

### Nucleic acid extraction

MERS-CoV viral RNA was prepared from infected Vero cell culture supernatants inactivated with 3:1 ratio of Trizol using the DirectZol RNA miniprep kit (Zymo Research, Irvine, CA, USA) according to the manufacturer’s protocol. The resulting viral RNA was used immediately and excess was stored at -80 °C. RNA was also extracted from similarly treated culture supernatants from uninfected Vero cells to serve as negative controls for the amplification assays.

Total nucleic acids were isolated from the NATtrol RP multimarker panels using the MasterPure Complete DNA and RNA Purification Kit (Epicentre, Madison, WI, USA) according to the manufacturer’s instructions.

## Supporting Information

S1 Fig Design and operation of real-time asymmetric five-primer OSD-LAMP. **A.** Schematic of fluorescent one-step strand displacement probes (OSD) for real-time sequence-specific signal transduction of LAMP. **B.** Schematic depicting positions of the OSD probe and the loop primer (LP) on the opposing loops of LAMP amplicons generated from MERS-CoV RNA.
(PDF)

S2 Fig Real-time fluorimetric functional analysis of MERS-CoV-specific LAMP primer sets. Cloned gBlock DNA surrogate MERS-CoV templates were amplified in standard four-primer LAMP assays containing EvaGreen. Input template copies are indicated next to each curve. The reactions were processed on LightCycler 96 and amplicons identity was validated by melting temperature analysis.
(PDF)

S3 Fig Limit of detection of asymmetric five-primer OSD-LAMP assays. Plasmids containing cloned DNA surrogates of MERS-CoV genomic targets located in ORF1a and ORF1b and in the region upstream of the gene E were used as templates for amplification. OSD-LAMP reactions were performed at 65 °C with 3 min incubations per cycle. The Cq of each amplification reaction as calculated by the LightCycler 96 software is tabulated.
(PDF)

S4 Fig Optimization of one-pot RT-LAMP assay for RNA detection using *in vitro* transcribed MERS-CoV target RNA. The primer set ORF1a.55 was used for optimizing the RT-LAMP-mediated amplification of 9 x 10^8^ copies of DNase I-treated synthetic ORF1a RNA. RT-LAMP reactions were performed at 65 °C with 3 min incubations per cycle on a LightCycler 96. Amplicon accumulation was measured in real-time as increase in fluorescence of EvaGreen.
(PDF)

S5 Fig Single mismatches with MERS-CoV template RNA do not compromise UpE.9 OSD-RT-LAMP assays. Parallel UpE.9 OSD-RT-LAMP assays were used to amplify none or various copies of the wild type or mutated MERS-CoV upE RNA targets. Synthetic template upE-mF1 was designed to mimic the T>C substitution observed at position 27427 in the MERS-CoV genomic sequence KJ156881.1. The upE-mLP template presents the C>T change located at position 8400 in the partial MERS-CoV genome KJ156873.1.
(PDF)
