# Supplementary material for: Real-Time Sequence-Validated Loop-Mediated Isothermal Amplification Assays for Detection of Middle East Respiratory Syndrome Coronavirus (MERS-CoV)
Source: PLoS One. 2015 Apr 9;10(4):e0123126. doi: 10.1371/journal.pone.0123126 (PMC4391951; doi:10.1371/journal.pone.0123126)
Supplement: S1 Fig — A. Schematic of fluorescent one-step strand displacement probes (OSD) for real-time sequence-specific signal transduction of LAMP. B. Schematic depicting positions of the OSD probe and the loop primer (LP) on the opposing loops of LAMP amplicons generated from MERS-CoV RNA. (PDF) [file pone.0123126.s001.pdf]

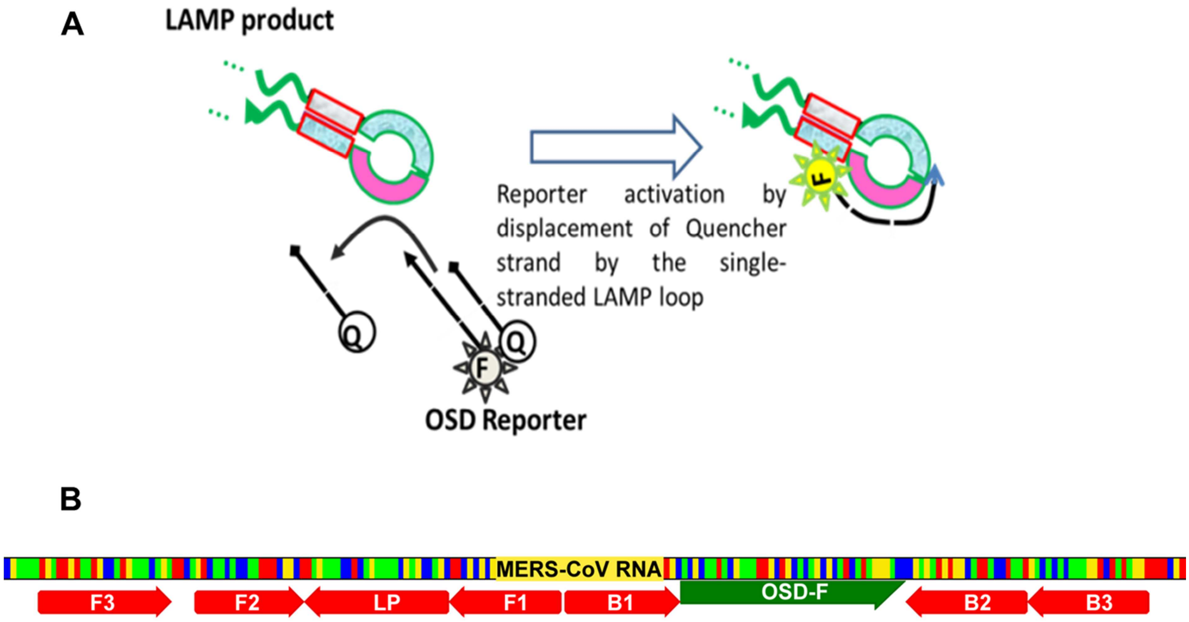

Supplementary Figure S1. Design and operation of real-time asymmetric five-primer OSD-LAMP. **A.** Schematic of fluorescent one-step strand displacement probes (OSD) for real-time sequence-specific signal transduction of LAMP. **B.** Schematic depicting positions of the OSD probe and the loop primer (LP) on the opposing loops of LAMP amplicons generated from MERS-CoV RNA.
