# Supplementary material for: Real-Time Sequence-Validated Loop-Mediated Isothermal Amplification Assays for Detection of Middle East Respiratory Syndrome Coronavirus (MERS-CoV)
Source: PLoS One. 2015 Apr 9;10(4):e0123126. doi: 10.1371/journal.pone.0123126 (PMC4391951; doi:10.1371/journal.pone.0123126)
Supplement: S2 Fig — Cloned gBlock DNA surrogate MERS-CoV templates were amplified in standard four-primer LAMP assays containing EvaGreen. Input template copies are indicated next to each curve. The reactions were processed on LightCycler 96 and amplicons identity was validated by melting temperature analysis. (PDF) [file pone.0123126.s002.pdf]

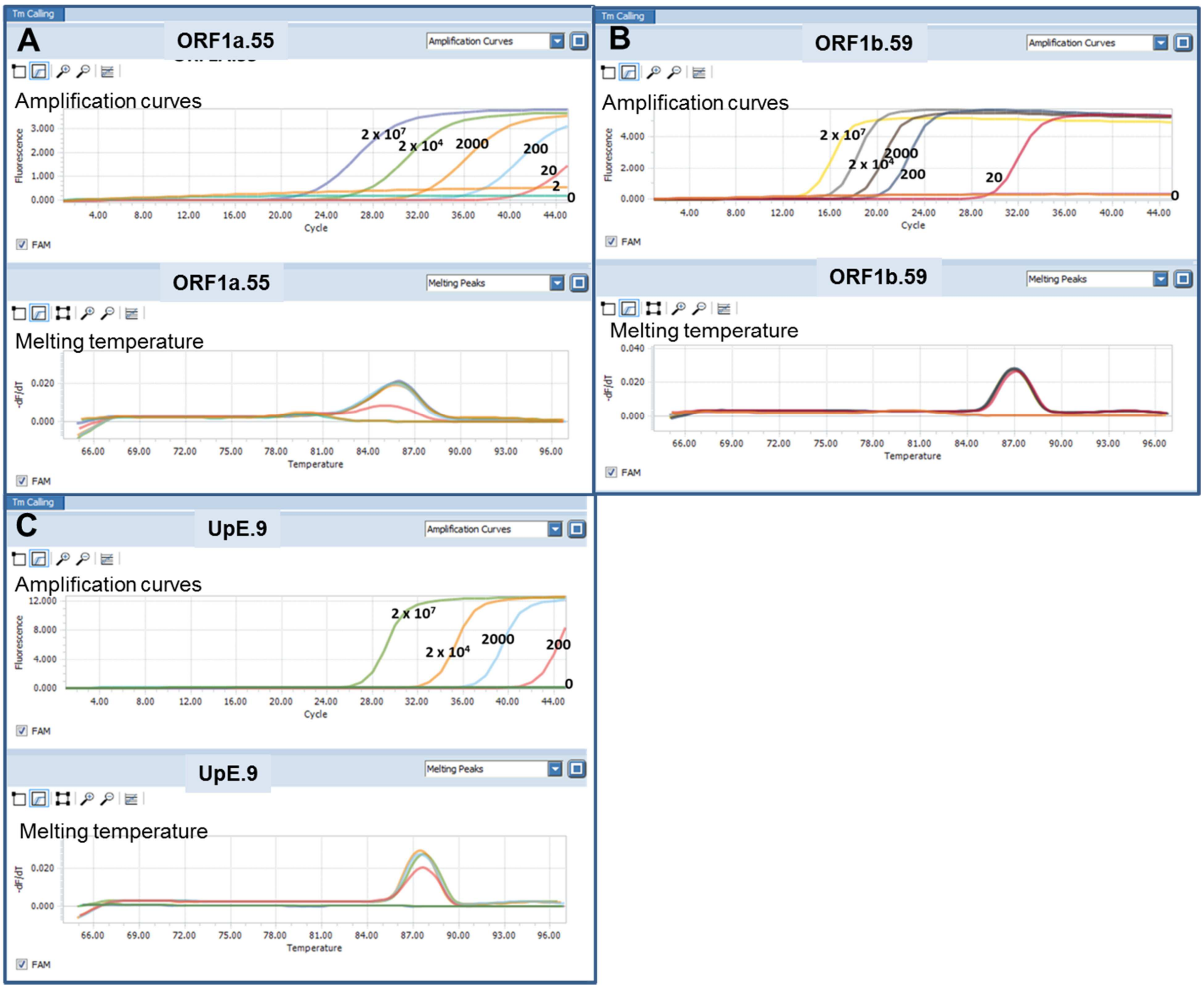

Supplementary Figure S2. Real-time fluorimetric functional analysis of MERS-CoV-specific LAMP primer sets. Cloned gBlock DNA surrogate MERS-CoV templates were amplified in standard four-primer LAMP assays containing the fluorescent intercalating dye EvaGreen. Input template copies are indicated next to each curve. The reactions were processed on LightCycler 96 and amplicons identity was validated by melting temperature analysis.
