# Supplementary material for: Real-Time Sequence-Validated Loop-Mediated Isothermal Amplification Assays for Detection of Middle East Respiratory Syndrome Coronavirus (MERS-CoV)
Source: PLoS One. 2015 Apr 9;10(4):e0123126. doi: 10.1371/journal.pone.0123126 (PMC4391951; doi:10.1371/journal.pone.0123126)
Supplement: S3 Fig — Plasmids containing cloned DNA surrogates of MERS-CoV genomic targets located in ORF1a and ORF1b and in the region upstream of the gene E were used as templates for amplification. OSD-LAMP reactions were performed at 65 °C with 3 min incubations per cycle. The Cq of each amplification reaction as calculated by the LightCycler 96 software is tabulated. (PDF) [file pone.0123126.s003.pdf]

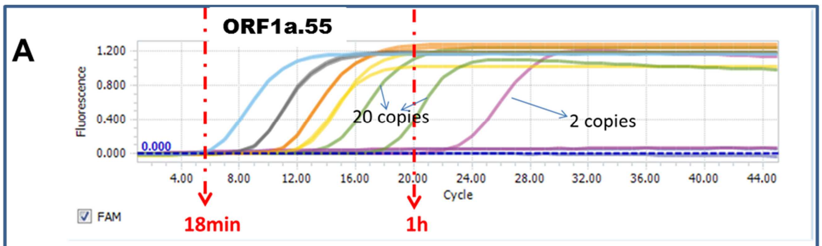

Color	Position ▲	Sample Name	Gene Name	Cq
(light blue)	B2	100pg-loop	None	5.94
(grey)	B3	1pg-loop	None	8.50
(orange)	B4	10fg-loop	None	10.72
(yellow)	B5	1fg-loop	None	11.97
(green)	B6	0.1fg	None	17.66 → 20 copies
(magenta)	B7	0.01fg	None	-
(blue)	B8	0	None	-

MERS-CoV ORF1a.55 OSD-LAMP assay can detect 20 copies within 54 min

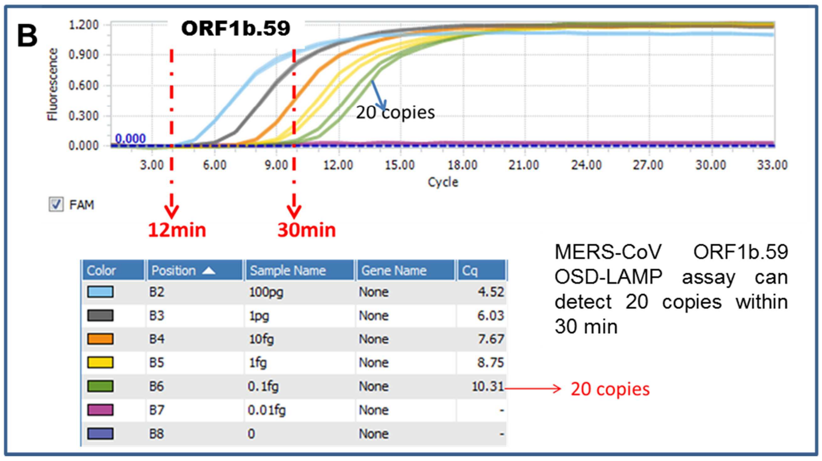

Color	Position ▲	Sample Name	Gene Name	Cq
(light blue)	B2	100pg	None	4.52
(grey)	B3	1pg	None	6.03
(orange)	B4	10fg	None	7.67
(yellow)	B5	1fg	None	8.75
(green)	B6	0.1fg	None	10.31 → 20 copies
(magenta)	B7	0.01fg	None	-
(blue)	B8	0	None	-

MERS-CoV ORF1b.59 OSD-LAMP assay can detect 20 copies within 30 min

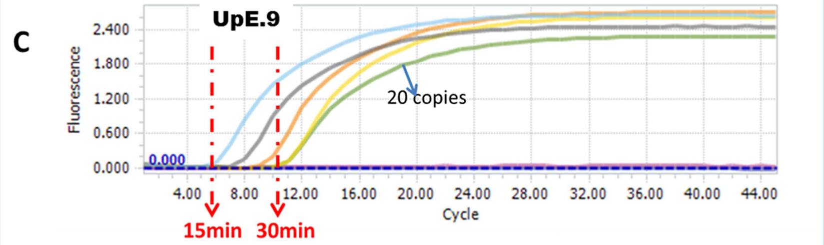

Color	Position ▲	Sample Name	Gene Name	Cq
(light blue)	B2	100pg-loop	None	5.40
(grey)	B3	1pg-loop	None	7.12
(orange)	B4	10fg-loop	None	8.90
(yellow)	B5	1fg-loop	None	10.33
(green)	B6	0.1fg-loop	None	10.29 → 20 copies
(magenta)	B7	0.01fg-loop	None	-
(blue)	B8	0-loop	None	-

MERS-CoV UpE.9 OSD-LAMP assay can detect 20 copies within 30 min

Supplementary Figure S3. Limit of detection of asymmetric five-primer OSD-LAMP assays for detection of plasmids containing cloned DNA surrogates of MERS-CoV genomic targets located in ORF1a and ORF1b and in the region upstream of the gene E. OSD-LAMP reactions were performed at 65 °C with 3 min incubations per cycle. The Cq of each amplification reaction as calculated by the LightCycler 96 software is tabulated.
