# Supplementary material for: Real-Time Sequence-Validated Loop-Mediated Isothermal Amplification Assays for Detection of Middle East Respiratory Syndrome Coronavirus (MERS-CoV)
Source: PLoS One. 2015 Apr 9;10(4):e0123126. doi: 10.1371/journal.pone.0123126 (PMC4391951; doi:10.1371/journal.pone.0123126)
Supplement: S4 Fig — The primer set ORF1a.55 was used for optimizing the RT-LAMP-mediated amplification of 9 x 10^8^ copies of DNase I-treated synthetic ORF1a RNA. RT-LAMP reactions were performed at 65 °C with 3 min incubations per cycle on a LightCycler 96. Amplicon accumulation was measured in real-time as increase in fluorescence of EvaGreen. (PDF) [file pone.0123126.s004.pdf]

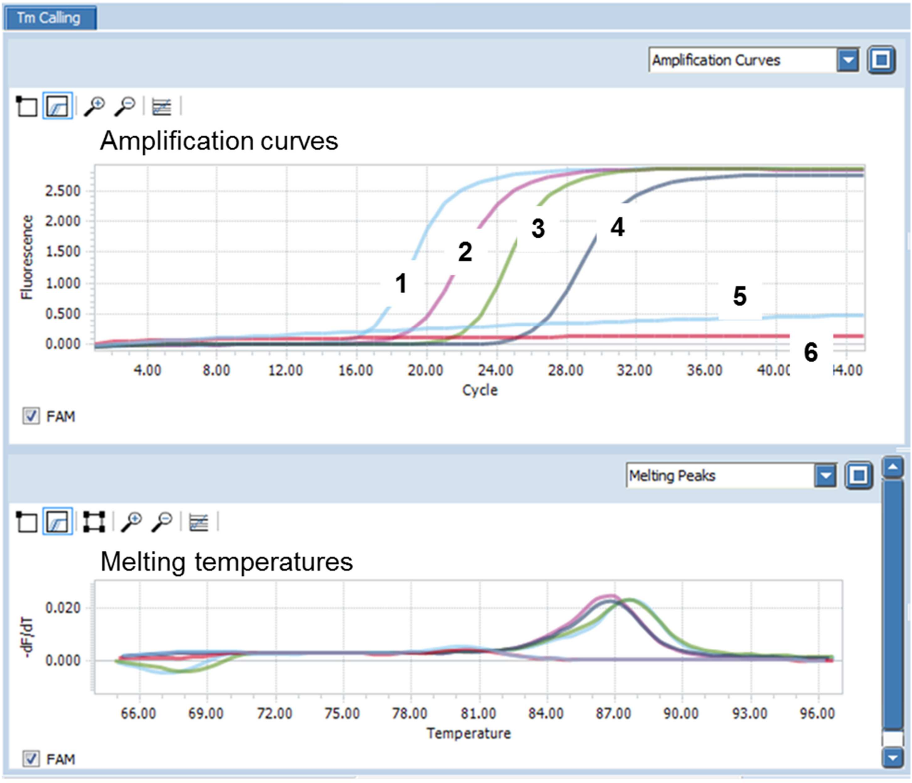

Sample	MERS-CoV ORF1A RNA	Bst 2.0	AMV RT	Betaine
1	9 x 10^8 copies	8 units	2 units	0.4 M
2	9 x 10^8 copies	8 units	2 units	1 M
3	9 x 10^8 copies	8 units	0	0.4 M
4	9 x 10^8 copies	8 units	0	1 M
5	0	8 units	2 units	1 M
6	0	8 units	2 units	0.4 M

Supplementary Figure S4. Optimization of one-pot RT-LAMP assay for RNA detection using *in vitro* transcribed MERS-CoV target RNA. The primer set ORF1a.55 was used for optimizing the RT-LAMP-mediated amplification of 9 x 10^8 copies of DNase I-treated synthetic ORF1a RNA. RT-LAMP reactions were performed at 65 °C with 3 min incubations per cycle on a LightCycler 96 real-time PCR machine. Amplicon accumulation was measured in real-time as increase in fluorescence of the intercalating dye EvaGreen.
