# Supplementary material for: Real-Time Sequence-Validated Loop-Mediated Isothermal Amplification Assays for Detection of Middle East Respiratory Syndrome Coronavirus (MERS-CoV)
Source: PLoS One. 2015 Apr 9;10(4):e0123126. doi: 10.1371/journal.pone.0123126 (PMC4391951; doi:10.1371/journal.pone.0123126)
Supplement: S5 Fig — Parallel UpE.9 OSD-RT-LAMP assays were used to amplify none or various copies of the wild type or mutated MERS-CoV upE RNA targets. Synthetic template upE-mF1 was designed to mimic the T>C substitution observed at position 27427 in the MERS-CoV genomic sequence KJ156881.1. The upE-mLP template presents the C>T change located at position 8400 in the partial MERS-CoV genome KJ156873.1. (PDF) [file pone.0123126.s005.pdf]

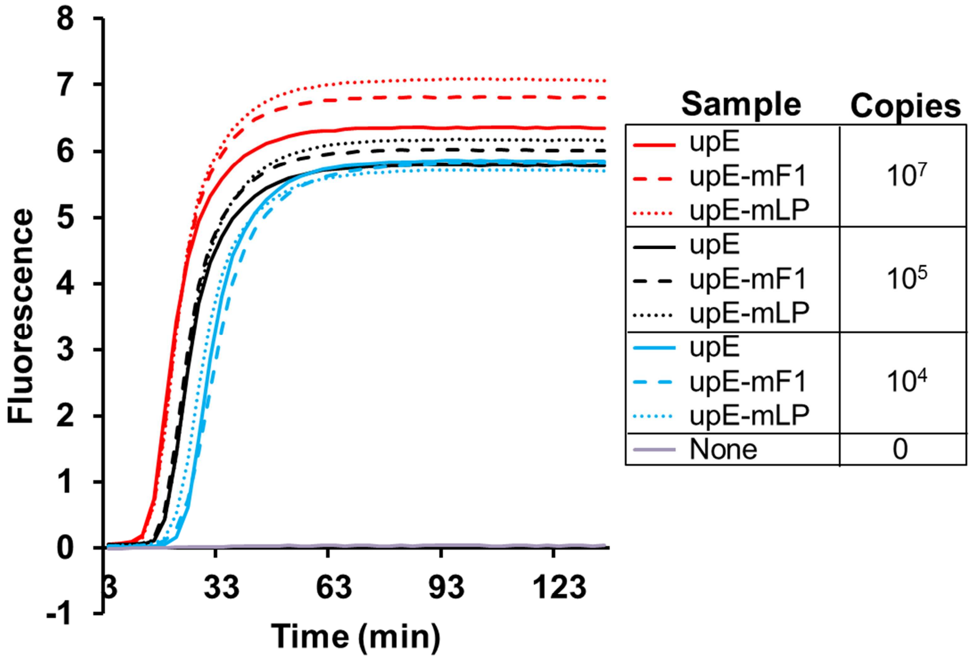

Supplementary Figure S5. Single mismatches with MERS-CoV template RNA do not compromise UpE.9 OSD-RT-LAMP assays. Parallel UpE.9 OSD-RT-LAMP assays were used to amplify none or various copies of the wild type or mutated MERS-CoV upE RNA targets. Synthetic template upE-mF1 was designed to mimic the T>C substitution observed at position 27427 in the MERS-CoV genomic sequence KJ156881.1. The upE-mLP template presents the C>T change located at position 8400 in the partial MERS-CoV genome KJ156873.1.
